# Methionine Synthase 2 Represses Stem Cell Maintenance of *Arabidopsis thaliana* in Response to Salt Stress

**DOI:** 10.3390/plants13162224

**Published:** 2024-08-10

**Authors:** Jiaqi Qiu, Minghuang Chen, Feng Lu, Xiaofen Chen, Zheqi Cai, Tao Huang

**Affiliations:** State Key Laboratory of Cellular Stress Biology, Xiamen Key Laboratory for Plant Genetics, School of Life Sciences, Xiamen University, Xiamen 361102, China; qiujq@stu.xmu.edu.cn (J.Q.); 21620221153373@stu.xmu.edu.cn (M.C.);

**Keywords:** salt stress, stem cell, AtMS2, signal transduction, *WUS/WOX*, meristem development

## Abstract

Salt stress represses the growth and development of plants that mainly depend on the continual propagation and differentiation of stem cells. WUSCHEL (WUS)/WUSCHEL-RELATED HOMEOBOX (WOX) family proteins determine stem cell fate in plants under ever-changing environments. It is not yet known how plant stem cell homeostasis is regulated under salt stress. Methionine synthase catalyzes the formation of methionine by methylating homocysteine in the one-carbon metabolism pathway. In this work, we investigated the role of *Arabidopsis* METHIONINE SYNTHASE 2 (AtMS2) in stem cell homeostasis under salt stress. The results showed that AtMS2 represses the stem cell maintenance of *Arabidopsis* in response to salt stress. Under normal growth conditions, AtMS2 is mainly localized in the cytoplasm. However, under salt stress, it exhibits significant accumulation in the nucleus. AtMS2 interacts with the WUS/WOX protein, and, together, they repress *WUS/WOX* expression by binding to its promoter. The mutation in *AtMS2* resulted in enhanced salt tolerance. Therefore, AtMS2 might act as a key negative regulator to repress the stem cell maintenance and growth of *Arabidopsis* under salt stress.

## 1. Introduction

The continual propagation and differentiation of totipotent stem cells provide an essential basis for the postembryonic development of plants. WUSCHEL (WUS) expressed in the organization center regulates stem cell propagation and activates *CLAVATA3* (*CLV3*) expression by moving to stem cell regions in the shoot apical meristem (SAM) [1,2,3]. Stem cell homeostasis in SAM is determined by a WUS-CLV3 feedback inhibition loop [4]. WUS protein regulates SAM development via the activation or repression of downstream genes by directly binding to different DNA motifs in the promoter region of these genes [5,6,7,8,9]. As another key shoot meristem regulator, SHOOT MERISTEMLESS (STM) is also involved in the WUS-CLV3 loop by interacting with WUS as a complex [10]. The WOX5 and WOX4 proteins of WUSCHEL-RELATED HOMEOBOX (WOX) family determine stem cell homeostasis in the root apical meristem (RAM) and vascular meristem (VM), respectively [11,12,13]. Plants have to face the ever-changing environment, which might be unfavorable for growth and development. Plants show developmental plasticity as an acclimation strategy via the modulation of their apical meristem activity after sensing the internal stress signal and external environmental stress [14,15,16,17]. High salt content in the soil is also a major environmental stress that represses plant growth and development. The response and developmental changes of plants to salt stress, as well as the underlying molecular mechanisms for salt perception and signaling, have been extensively investigated in recent decades [18,19,20]. It was reported that salt stress represses root growth by decreasing root meristem activity [21]. Salt stress decreases root meristem size by triggering the accumulation of nitric oxide, which further reduces auxin levels by decreasing PINFORMED (PIN) expression and represses auxin signaling by stabilizing INDOLE-3-ACETIC ACID17 (IAA17) [22]. Under salt stress, the activated SALT OVERLY SENSITIVE (SOS) signaling pathway also maintains meristem activity and primary root growth by stabilizing APETALA2 (AP2)-domain transcription factor PLETHORAs (PLT1 and PLT2) [23]. The condensation of STM in the nucleus is also required for maintaining the shoot meristem; moreover, nuclear STM condensation is stimulated by salt stress and allows for enhanced salt tolerance [24]. So far, the mechanism employed for stem cell maintenance in different apical meristems under salt stress is still largely unknown.

Methionine (Met) is essential for the synthesis of proteins and the production of cellular *S*-adenosylmethionine, with the latter acting as the universal activated methyl donor for the majority of the methylation process of DNA, RNA, proteins, and metabolites [25]. In *Arabidopsis*, three cobalamin-independent methionine synthase isoforms including cytosolic AtMS1 (AT5G17920), cytosolic AtMS2 (AT3G03780), and plastid AtMS3 (AT5G20980) catalyze the regeneration of Met from homocysteine in the one-carbon metabolism pathway [26]. *AtMS1* mutation causes a lower cellular Met level and reduces *S*-adenosylmethionine accessibility to DNA or histone methyltransferases, resulting in a significant reduction in the genome-wide DNA methylation level [27]. Mutations in *AtMS2* or *AtMS3* only cause a slight reduction in cellular Met content and have no effect on the genome-wide DNA methylation level [27]. In *Arabidopsis*, *S*-adenosylmethionine is predominantly synthesized by methionine adenosyltransferase 4/S-adenosyl-Met synthetase 3 (MAT4/AtSAMS3) using Met and ATP as substrates. The *MAT4*/*AtSAMS3* mutation causes a reduction in cellular *S*-adenosylmethionine content and decreases the methylation level of DNA and histone [28]. It was also reported that the *MAT4*/*AtSAMS3* mutation causes *Arabidopsis* to be hypersensitive to salt stress, while the overexpression of *S-adenosyl-Met synthetase 2* from sugar beet increases the tolerance of Arabidopsis to salt and oxidative stress [29]. It seems that the one-carbon metabolism pathway plays a pivotal role in plant salt tolerance by regulating the genome-wide DNA methylation level. It remains to be elucidated whether the one-carbon metabolism pathway can regulate *WUS/WOX* expression and apical meristem activity under salt stress.

In our previous work, we identified AtMS2 as a possible *WUS* promoter-binding protein through a DNA pull-down assay using biotin-labeled *WUS* promoter fragments followed by mass spectrometry identification [30]. A possible link was indicated between the one-carbon metabolism pathway and stem cell maintenance. Therefore, the role of AtMS2 in stem cell maintenance under salt stress was further examined in this work. Our results showed that AtMS2 might repress *WUS/WOX* expression and stem cell maintenance of plants under salt stress.

## 2. Results

### 2.1. AtMS2 Binds to the WUS/WOX Promoter

**Figure 1:**
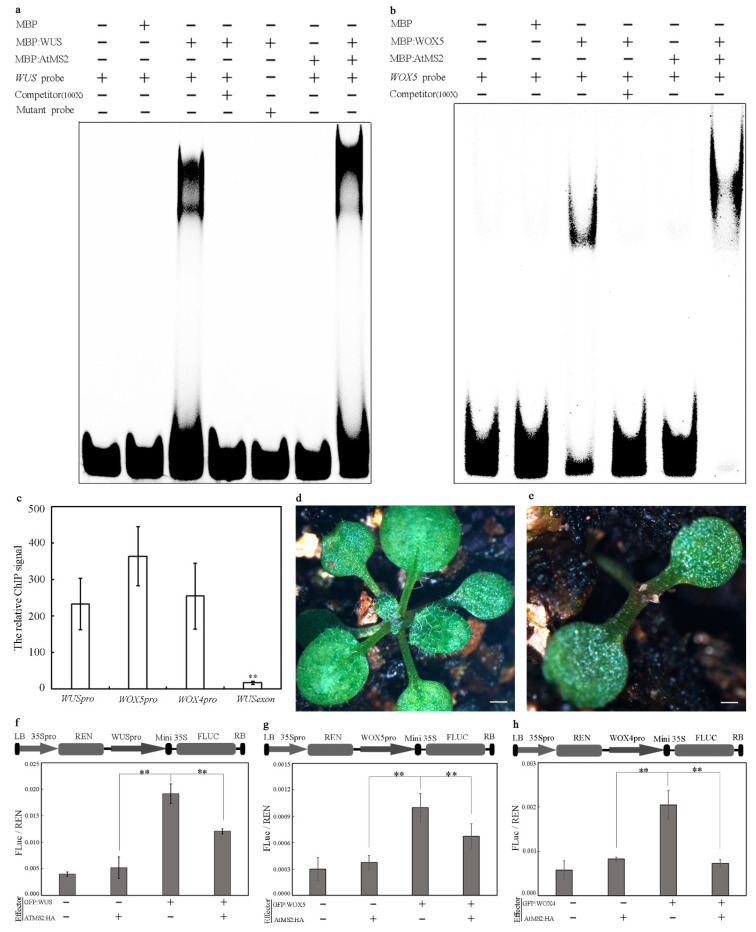
AtMS2 binds to the *WUS/WOX* promoter. (**a**,**b**) MBP:AtMS2 binds to a Texas red-labeled *WUS* promoter probe (**a**) and a *WOX5* promoter probe (**b**) in the presence of MBP:WUS and MBP:WOX5 in the EMSA assay. The unlabeled probes were used as competitors. The *WUS* probe and *WOX5* probe are located at −837–−879 bp and −393–−435 bp in the *WUS* promoter and *WOX5* promoter, respectively. The TAAT motif in *WUS* mutant probe was replaced by GGGG. (**c**) Chromatin immunoprecipitation of AtMS2:HA with the *WUS/WOX* promoter in 3-day-old *35S_pro_::AtMS2:HA* plant. The regions containing the TAAT motif in *WUS* promoter (−746–−908 bp), *WOX5* promoter (−494–−810 bp), and *WOX4* promoter (−1020–−1256 bp) were analyzed, respectively. An exon region of the *WUS* gene (+1421–+1547 bp) was examined in a control assay. Error bar = mean ± SD (n = 4 replicates). Student’s *t*-test. ** *p* < 0.01. (**d**,**e**) The growth phenotype of 3-week-old WT (d) and one *35S_pro_::AtMS2:HA* line showing growth cessation (**e**). Scale bar = 0.5 mm. (**f**–**h**) FLuc/REN ratio in *Nicotiana benthamiana* epidermal cells with transient expression of *WUS_pro_::LUC* (**f**), *WOX5_pro_::LUC* (**g**), and *WOX4_pro_::LUC* (**h**) dual luciferase reporters, respectively, in combination with indicated effectors. 35S: CaMV 35S promoter; Mini 35S: a 47-base-pair 35S minimal element; REN: renilla luciferase, FLUC: firefly luciferase. LB/RB: T-DNA left or right border. Error bar = mean ± SD (*n* = 4). Student’s *t*-test. ** *p* < 0.01.

Both the WUS and WOX5 proteins were previously reported to form protein complexes with other proteins, which further activate *WUS/WOX* expression by binding to the TAAT motif in the *WUS/WOX* promoter [30,31,32]. Since the AtMS2 protein was identified as a possible *WUS* promoter-binding protein [30], we further examined whether AtMS2 might also bind to the *WUS*/*WOX* promoter by interacting with WUS/WOX5 using the electrophoretic mobility shift assay (EMSA). The purified MBP (maltose-binding protein):WUS fusion protein bound to Texas red-labeled *WUS* promoter probes containing a TAAT motif, but not to mutant probes with the TAAT motif substituted by GGGG (Figure 1a). MBP:AtMS2 alone failed to bind to the *WUS* promoter probe, but caused a super shift of the *WUS* promoter probe in the presence of the MBP:WUS protein (Figure 1a), suggesting that AtMS2 might bind to the *WUS* promoter probe by interacting with WUS. Consistently, MBP:AtMS2 also caused a super shift of a Texas red-labeled *WOX5* promoter probe in the presence of the MBP:WOX5 protein in an EMSA assay (Figure 1b). These results demonstrated that AtMS2 can bind to the *WUS/WOX5* promoter by interacting with WUS/WOX5. The binding of AtMS2 to the *WUS/WOX* promoter was further confirmed by a Chromatin immunoprecipitation (ChIP) experiment using 3-day-old *35S_pro_::AtMS2:HA* seedling expressing the HA-tagged AtMS2 protein. The abundance of the examined regions in the *WUS*, *WOX5*, and *WOX4* promoters, but not the control exon region of the *WUS* gene, was significantly increased in the ChIP analysis (Figure 1c). Therefore, the AtMS2 protein might bind to the *WUS*, *WOX5*, and *WOX4* promoters in Arabidopsis. In addition, the growth and leaf differentiation of some *35S_pro_::AtMS2:HA* lines were completely compressed after germination, demonstrating that the overexpression of *AtMS2* might repress the growth and development of *Arabidopsis* (Figure 1d,e). The effects of the AtMS2 protein on the activity of the *WUS/WOX* promoter were further examined in *Nicotiana benthamiana* leaf epidermal cells using dual-luciferase assays. GFP:WUS and AtMS2:HA proteins were transiently expressed in *Nicotiana benthamiana* leaf epidermal cells, and luciferase reporter activity in *WUS_pro_::LUC* was measured. Luciferase activity was significantly increased in the presence of the GFP:WUS protein, suggesting that WUS binds to and activates the *WUS* promoter. On the other hand, luciferase activity decreased significantly when GFP:WUS and AtMS2:HA were co-expressed (Figure 1f). Similar results were obtained when GFP:WOX5 or GFP:WOX4 were co-expressed with AtMS2:HA and when the activity of *WOX5* or *WOX4* promoter was measured (Figure 1g,h). These results demonstrated that AtMS2 might repress *WUS/WOX* expression by forming a protein complex with the WUS/WOX protein and by binding to the *WUS/WOX* promoter.

### 2.2. AtMS2 Interacts with WUS/WOX as a Complex

**Figure 2:**
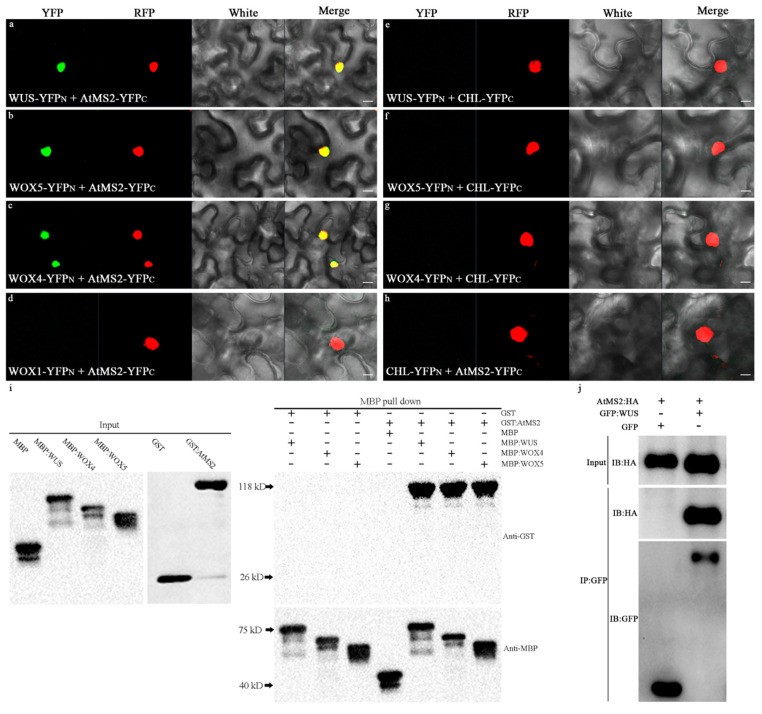
AtMS2 interacts with WUS/WOX in the nucleus. (**a**–**h**) Bimolecular fluorescence complementation in *Nicotiana benthamiana* epidermal cells. AtMS2:YFP_C_ protein in combination with WUS:YFP_N_ (**a**), WOX5:YFP_N_ (**b**), and WOX4:YFP_N_ (**c**), respectively, was colocalized with Coilin:RFP protein in the nucleus. WOX1:YFP_N_, Chloramphenicol Acetyltransferase (CHL):YFP_N_, and CHL:YFP_C_ were used in control assays (**d**–**h**). Scale bar = 25 µm. (**i**) Input of recombinant proteins detected by immunoblotting with anti-GST antibody and anti-MBP antibody (Left) and pull-down of AtMS2 in fusion with GST through MBP-tagged WUS/WOX protein (Right). (**j**) AtMS2 interacts with GFP:WUS, but not GFP in Nicotiana benthamiana epidermal cells in a co-immunoprecipitation assay.

To further examine the interaction between the AtMS2 and WUS/WOX proteins, a bimolecular fluorescence complementation (BiFC) assay was performed in leaf epidermal cells of *Nicotiana benthamiana*. The Coilin:RFP protein was transiently expressed in epidermal cells as a nucleus marker [33]. Strong fluorescence of YFP and RFP was observed in the nucleus of many epidermal cells when AtMS2 in fusion with the C-terminal half of YFP (AtMS2-YFP_C_) and WUS in fusion with the N-terminal half of YFP (WUS-YFP_N_), as well as Coilin:RFP protein, were transiently co-expressed in *Nicotiana benthamiana* epidermal cells (Figure 2a). Therefore, AtMS2 might interact with WUS in the nucleus. AtMS2 also interacted with WOX5 and WOX4 in the nucleus of leaf epidermal cells in the BiFC assay (Figure 2b,c), while it failed to interact with WOX1, which is another member of the WOX family (Figure 2d). YFP fluorescence was not observed in negative control assays either (Figure 2e–h). The interaction between AtMS2 and WUS/WOX was further examined by an in vitro pull-down assay. The results showed that the purified AtMS2 protein fused with GST can directly interact with MBP:WUS, MBP:WOX5, and MBP:WOX4 proteins but not with the MBP protein (Figure 2i). In addition, the AtMS2 protein was demonstrated to interact with the WUS protein in *Nicotiana benthamiana* epidermal cells in a co-immunoprecipitation assay (Co-IP) (Figure 2j). Taken together, the above results demonstrated that AtMS2 can directly interact with WUS, WOX5, and WOX4.

### 2.3. Salt Stress Regulates the Expression and Subcellular Localization of AtMS2

**Figure 3:**
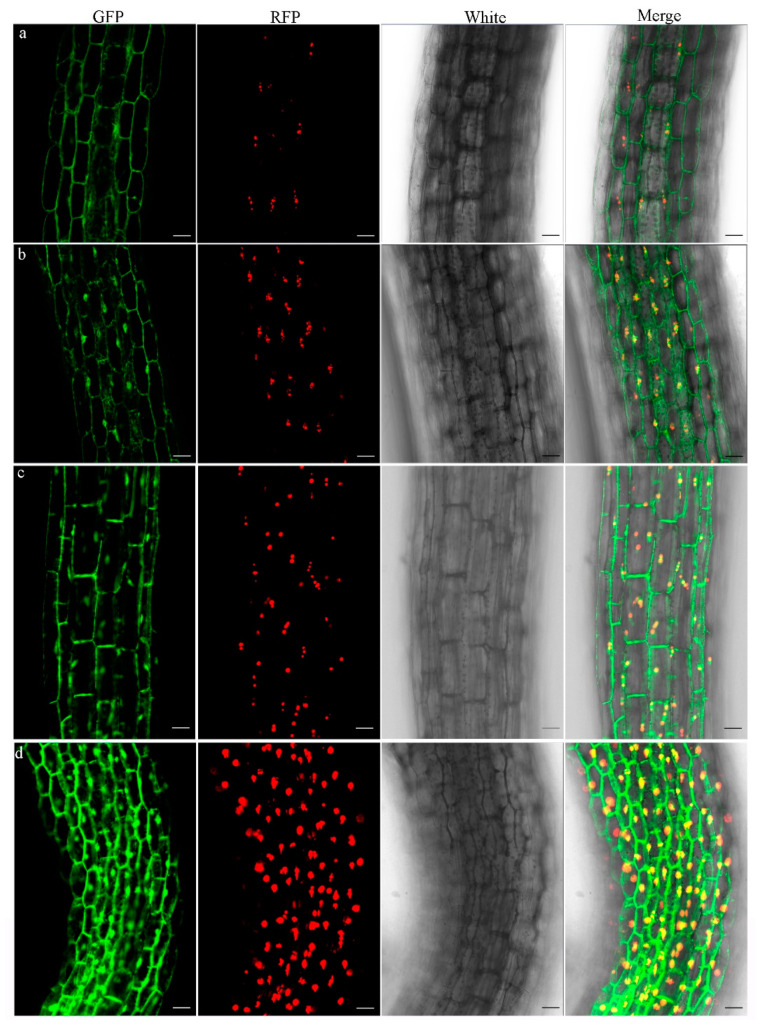
Salt stress increases *AtMS2* expression and induces the accumulation of AtMS2 protein in the nucleus. (**a**,**b**) AtMS2:GFP was accumulated in the nucleus of 5-day-old *35S_pro_::AtMS2:GFP 35S_pro_::Coilin:RFP* plants grown on MS medium containing 80 mM NaCl (**b**) in comparison with control plants (**a**). (**c**,**d**) AtMS2:GFP:GUS expression level was increased in 5-day-old *gAtMS2::GFP:GUS* plants grown on MS medium containing 120 mM NaCl (**d**) in comparison with control plants (**c**). Scale bar = 200 µm.

To further examine the subcellular localization of AtMS2 protein in response to salt stress, the *35S_pro_::AtMS2:GFP* construct was generated and directly introduced into *35S_pro_::Coilin:RFP* transgenic plants. AtMS2:GFP was mainly observed in the cytoplasm in 5-day-old *35S_pro_::AtMS2:GFP 35S_pro_::Coilin:RFP* seedlings grown on MS media (Figure 3a). However, AtMS2:GFP clearly accumulated in the nucleus in 5-day-old *35S_pro_::AtMS2:GFP 35S_pro_::Coilin:RFP* seedlings grown on media supplemented with 80 mM of NaCl (Figure 3b), suggesting that salt stress can trigger the accumulation of AtMS2 in the nucleus. Salt stress causes osmotic stress and an increase in the abscisic acid (ABA) content of plants [34,35,36]. Salt stress also causes the flooding of reactive oxygen species (ROS), which act as a signaling molecule to trigger downstream gene expression [37]. The subcellular localizations of AtMS2:GFP protein were further examined in a *35S_pro_::AtMS2:GFP* plant subjected to sorbitol, ABA, or H_2_O_2_ treatment. The results showed that the accumulation of AtMS2:GFP protein in the nucleus can be triggered by 0.6 M sorbitol or 2 mM H_2_O_2_ treatment, but not by 180 μM ABA treatment (Figure S1). Therefore, the accumulation of AtMS2 in the nucleus might result from the osmotic stress and/or ROS accumulation triggered by salt stress.

To further examine the expression pattern of *AtMS2*, a 7234 bp genomic sequence of the *AtMS2* gene including a 2841 bp promoter region, 3139 bp coding region, and 1253 bp 3′downstream region was cloned to create *gAtMS2::GFP:GUS* plants that express the AtMS2:GFP:GUS fusion protein under the control of the *AtMS2* promoter. GUS staining showed that AtMS2:GFP:GUS is expressed in young seedling, shoots, and all floral organs in flowers after the germination of several independent lines (Figure S2). When 5-day-old *gAtMS2::GFP:GUS* seedlings were germinated and grown on MS medium containing NaCl, the expression level of the AtMS2:GFP:GUS protein was clearly increased in comparison with that in control seedlings (Figure 3c,d). Furthermore, AtMS2:GFP:GUS protein also clearly accumulated in the nucleus of root cells under salt stress (Figure 3d). Therefore, salt stress promotes the expression of the *AtMS2* gene and the nuclear accumulation of AtMS2 protein.

### 2.4. AtMS2 Represses WUS/WOX Expression under Salt Stress

**Figure 4:**
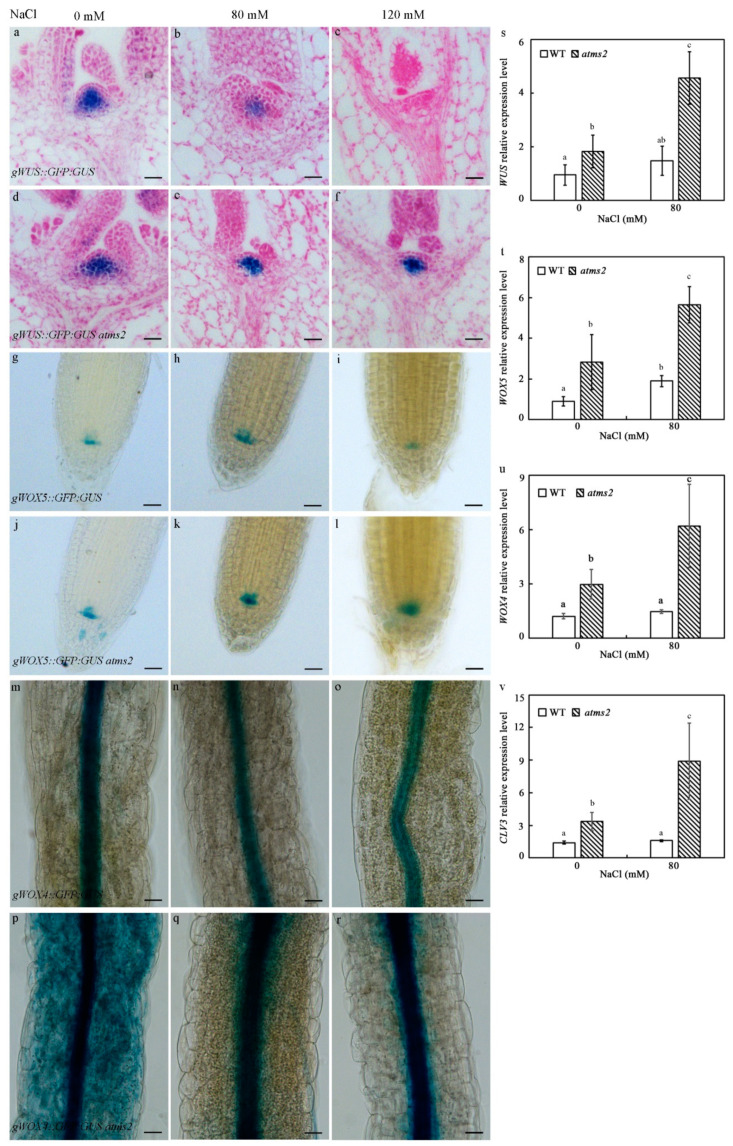
AtMS2 represses *WUS/WOX* expression under salt stress. (**a**–**f**) The expression of WUS:GFP:GUS protein is decreased to a lower level in 7-day-old *gWUS::GFP:GUS* seedlings (**a**–**c**) in comparison with *gWUS::GFP:GUS atms2* seedlings (**d**–**f**) grown on media containing 80 mM (**b**,**c**) and 120 mM NaCl (**c**,**f**), respectively. (**g**–**l**) WOX5:GFP:GUS protein is expressed at a higher level in 7-day-old *gWOX5::GFP:GUS atms2* seedlings (**j**–**l**) in comparison with *gWOX5::GFP:GUS* seedlings (**g**–**i**) grown on media containing 80 mM (**h**,**k**) and 120 mM (**i**,**l**) NaCl, respectively. (**m**–**r**) WOX4:GFP:GUS protein is expressed at a higher level in 7-day-old *gWOX4::GFP:GUS atms2* seedlings (**p**–**r**) in comparison with *gWOX4::GFP:GUS* seedlings (**m**–**o**) grown on media containing 80 mM (**n**,**q**) and 120 mM (**o**,**r**) NaCl, respectively. Scale bar = 50 µm (**a**–**f**), 25 µm (**g**–**l**), and 200 µm (**m**–**r**). (**s**–**v**) The RT-qPCR analysis of *WUS* (**s**), *WOX5* (**t**), *WOX4* (**u**), and *CLV3* (**v**) mRNA in 5-day-old WT and *atms2* mutant grown on medium containing 80 mM NaCl. *Actin2* was examined as a reference gene. Error bar = mean ± SD (*n* = 3). Statistical differences (*p* ≤ 0.01) between different plants were determined by one-way ANOVA followed by Tukey’s multiple comparison test.

To explore the role of the *AtMS2* gene in regulating the expression of *WUS* in *Arabidopsis* under salt stress, an *atms2* knockout mutant was identified by reverse transcription polymerase chain reaction (RT-PCR) analysis (Figure S3). The *gWUS::GFP:GUS* plant was used to create *gWUS::GFP:GUS atms2* plants via genetic crossing. GUS staining showed that the protein level of WUS:GFP:GUS was clearly decreased in 7-day-old *gWUS::GFP:GUS* plants grown on media containing NaCl at different concentrations (Figure 4a–c). Consistently, the SAM of WT was flattened in the presence of 120 mM NaCl (Figure 4c). However, WUS:GFP:GUS was expressed at a relatively higher level in *gWUS::GFP:GUS atms2* plants in comparison with *gWUS::GFP:GUS* plants when grown on media containing NaCl (Figure 4d–f). To explore the roles of *AtMS2* in regulating the expression of WOX5 and WOX4 proteins in Arabidopsis under salt stress, *gWOX5::GFP:GUS atms2* and *gWOX4::GFP:GUS atms2* plants were also created by crossing. WOX5:GFP:GUS and WOX4:GFP:GUS proteins were also expressed at a higher level in *gWOX5::GFP:GUS atms2* and *gWOX4::GFP:GUS atms2* plants in comparison with *gWOX5::GFP:GUS* and *gWOX4::GFP:GUS* plants, respectively, upon NaCl treatment (Figure 4g–r). RT-qPCR analysis also showed that the expression level of *WUS*, *WOX5*, *WOX4*, and *WUS* downstream target *CLV3* in 5-day-old *atms2* mutant was already higher than that in WT under normal growth conditions, demonstrating that the nuclear localization of AtMS2 and the repressive effect of AtMS2 on *WUS/WOX* expression can be observed even under normal growth conditions. In the presence of 80 mM of NaCl, the expression of *WUS*, *WOX5*, *WOX4*, and *CLV3* in 5-day-old *atms2* mutant increased to a higher level in comparison with that of the 5-day-old WT seedlings (Figure 4s–v), demonstrating the predominant repression of *WUS/WOX* expression by AtMS2 under salt stress. In addition, the expression of *WUS* and *WOX4* mRNA in WT seedlings was not changed, while *WOX5* mRNA in WT seedlings was also slightly increased in the presence of 80 mM NaCl, demonstrating that in response to salt stress, the expression of *WUS/WOX* might be regulated at transcriptional and post-transcriptional levels by many promotive and inhibitory factors, including AtMS2. Taken together, AtMS2 represses the expression of the *WUS/WOX* gene when it is subjected to salt stress.

### 2.5. AtMS2 Represses Plant Growth and Development under Salt Stress

**Figure 5:**
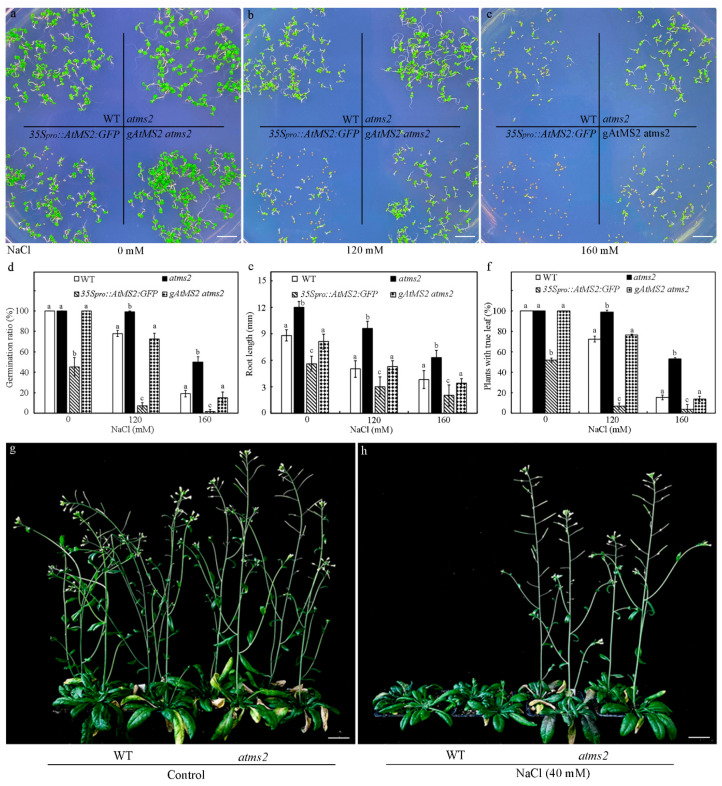
AtMS2 represses the growth of plants under salt stress. (**a**–**c**) The growth phenotype of 7-day-old WT, *35S_pro_::AtMS2:GFP*, *atms2*, and *gAtMS2 atms2* seedlings on media containing NaCl at different concentrations. (**d**–**f**) The germination ratio (**d**), root length (**e**), and percentage of plants with true leaf differentiation (**f**) of 7-day-old WT, *35S_pro_::AtMS2:GFP*, *atms2*, and *gAtMS2 atms2* seedlings on media containing NaCl at different concentrations. Error bar = mean ± SD (*n* = 3). Statistical differences (*p* ≤ 0.01) between different plants were determined by one-way ANOVA followed by Tukey’s multiple comparison test. (**g**,**h**) The growth phenotype of 2-month-old WT and *atms2* mutant grown on soil under salt stress (**h**) in comparison with control plants (**g**). Scale bar = 1.0 cm (**a**–**c**) and 2.0 cm (**g**,**h**).

The same 7.324 Kb *AtMS2* genomic sequence used for the *gAtMS2::GFP:GUS* construct was also used to create the *gAtMS2* construct, which was introduced into the *atms2* mutant for the complementation test. The germination and growth of 7-day-old WT, *atms2*, *gAtMS2 atms2*, and *35S_pro_::AtMS2:GFP* seedlings on media containing NaCl at different concentrations were examined. After sowing on the control MS medium for 7 days, the germination ratio of the *35S_pro_::AtMS2:GFP* seed was clearly lower than that of the WT, *atms2*, and *gAtMS2 atms2* seeds (Figure 5a,d), suggesting the nuclear localization of AtMS2:GFP protein even under normal growth conditions. On the MS medium containing 120 mM NaCl, the germination ratio of the *35S_pro_::AtMS2:GFP* seedling was more strongly reduced in comparison with other seedlings, suggesting that the overexpression of AtMS2 protein causes hypersensitivity to salt stress (Figure 5b,d). On the medium containing 160 mM of NaCl, the germination ratio of *atms2* was higher than that of WT and *gAtMS2 atms2*, demonstrating that *atms2* was insensitive to salt stress (Figure 5c,d). When 2-day-old WT, *atms2*, *gAtMS2 atms2*, and *35S_pro_::AtMS2:GFP* seedlings grown on control MS media were transferred to MS media containing NaCl at different concentrations for another 5 days, *atms2* mutants had longer primary roots and a higher percentage of seedlings with true leaves than that of the WT and *gAtMS2 atms2* seedlings, while the *35S_pro_::AtMS2:GFP* seedling exhibited the opposite phenotypes (Figure 5e,f and Figure S4). These results demonstrated that AtMS2 represses plant growth and development under salt stress. To examine the effects of long-term NaCl treatment on the growth and development of the *atms2* mutant, the WT and *atms2* mutant were grown on soil and watered with 40 mM NaCl once per week. After a two-month NaCl treatment, the inflorescent shoot development of WT was clearly suppressed, while the inflorescent shoot of the *atms2* mutant grew up and produced flowers as those of the control plants under normal growth conditions (Figure 5g,h). Therefore, the loss of function of *AtMS2* enhanced the salt tolerance of Arabidopsis.

#### AtMS2 Mutation Does Not Affect Met Content under Salt Stress

**Figure 6:**
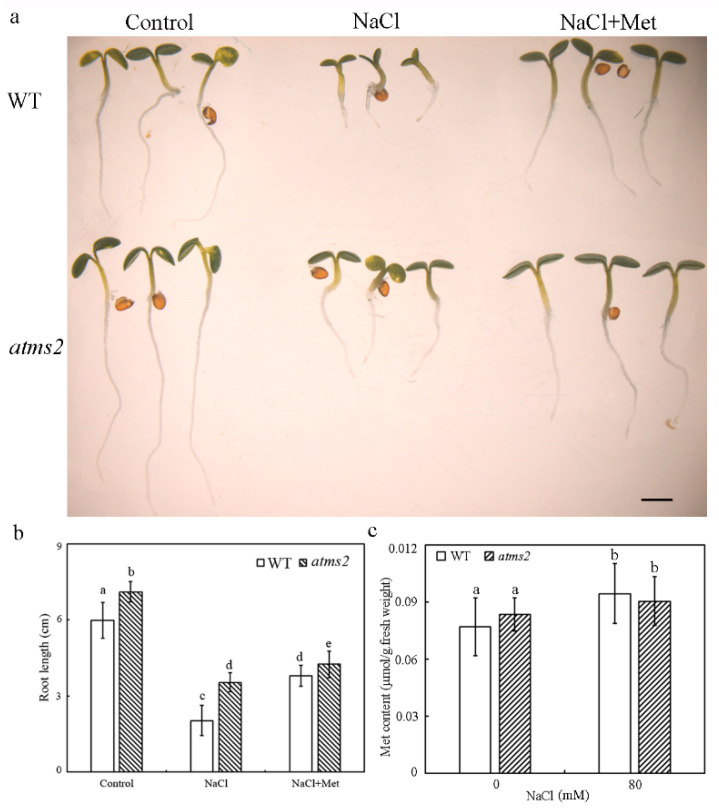
Met treatment enhances salt tolerance of Arabidopsis. (**a**,**b**) The growth phenotype and root length of 5-day-old WT and the *atms2* mutant on MS medium containing 80 mM NaCl with or without 1 mM Met. Scale bar = 1 mm. (**c**) The Met content of 5-day-old WT and *atms2* mutant grown on MS medium in the presence or absence of 80 mM NaCl. Error bar = mean ± SD (*n* = 3). Statistical differences (*p* ≤ 0.05) were determined by one-way ANOVA followed by Tukey’s multiple comparison test.

Met is converted into *S*-adenosylmethionine by methionine adenosyltransferase 4 in the one-carbon metabolism pathway, and cellular *S*-adenosylmethionine content further determines the salt tolerance of Arabidopsis by regulating the genome-wide DNA methylation level [28,29]. The effect of Met treatment on the salt tolerance of WT and the *atms2* mutant were further examined. The results showed that the addition of 1 mM Met to the medium significantly increases the root length of WT and the *atms2* mutant grown on the medium containing 80 mM NaCl, suggesting that Met can enhance the salt tolerance of both WT and *atms2* (Figure 6a,b). It was reported that the *AtMS2* mutation only caused a slight reduction in cellular Met content under normal growth conditions [27]. In this work, cellular Met content was further examined in WT and the *atms2* mutant subjected to NaCl treatment. Actually, Met content was slightly increased in WT and the *atms2* mutant in the presence of 80 mM NaCl, while there was no significant difference between WT and the *atms2* mutant (Figure 6c). Taken together, although Met can enhance the salt tolerance of Arabidopsis, the AtMS2 protein might cause the repression of growth and development under salt stress. Therefore, the *atms2* mutant had the same Met content as WT under salt stress but showed enhanced salt tolerance in comparison with WT.

## 3. Discussion

The stem cell homeostasis of SAM is maintained by a WUS-CLV3 feedback inhibition loop [4]. The secreted CLV3 peptide can bind to its receptors, including the leucine-rich repeat receptor-like kinases (LRR-RLK) CLV1/2, CORYNE, BARELY ANY MERISTEM1 (BAM1), and CLAVATA3 INSENSITIVE RECEPTOR KINASES (CIKs), on the plasma membrane, resulting in the final downregulation of *WUS* expression [4,38,39,40]. It has been revealed that a *clv3* mutant and a *clv1 bam1* double mutant exhibit the tolerant phenotype of SAM development to salt stress, which is independent of typical stress-responsive genes [41]. Since the loss of function of *CLV3* or the interruption of its signaling pathway definitely enhances *WUS* expression, these results indicate a possibility that a high level of *WUS* expression might increase SAM tolerance to salt stress by promoting stem cell maintenance. So far, it still remains largely unknown how salt signaling is involved in the WUS-CLV3 framework during SAM acclimation to salt stress. More work is still required to explore the interaction between salt stress and stem cells.

**Figure 7:**
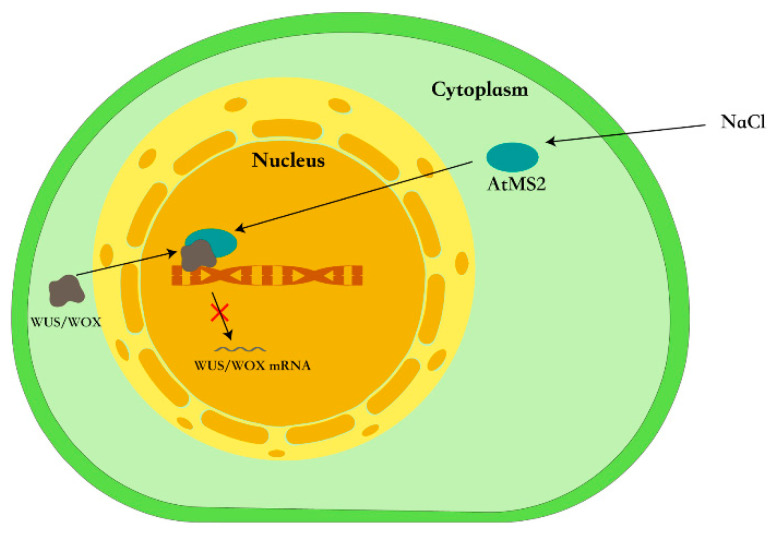
A proposed model for the repression of *WUS/WOX* gene by AtMS2 in response to salt stress. Salt stress induces the nuclear translocation of AtMS2, which further binds to the *WUS/WOX* promoter and represses *WUS/WOX* expression by interacting with WUS/WOX.

In this work, the results show that salt stress can increase the expression of *AtMS2* and promote the accumulation of AtMS2 in the nucleus (Figure 3). AtMS2 can interact with WUS/WOX protein and further represses the expression of the *WUS/WOX* gene by binding to its promoter (Figure 2 and Figure 4). Consistently, the *atms2* mutant has a higher *WUS/WOX* expression level under salt stress and shows enhanced salt tolerance, while the overexpression of *AtMS2* causes hypersensitivity of plants to salt stress (Figure 5). These results demonstrate that AtMS2 can be recruited into a transcriptional complex that represses *WUS/WOX* expression and plant growth under salt stress. Taken together, our signaling model proposes that AtMS2 might act as a negative regulator to repress meristem activity in response to salt stress (Figure 7).

Both *AtMS1* and *AtMS2* encode a cytosolic methionine synthase, while only the *AtMS1* mutation significantly reduces cellular Met content and the DNA methylation level [27]. This might be the reason that the complete loss of function of *AtMS1* causes the lethal phenotype but not that of *AtMS2* [27]. In Arabidopsis, Met is further converted into *S*-adenosylmethionine by MAT4/AtSAMS3, and the *MAT4*/*AtSAMS3* mutation also reduces cellular *S*-adenosylmethionine content and the methylation level of DNA and histones [28]. The *MAT4*/*AtSAMS3* mutation also causes the hypersensitivity of Arabidopsis to salt stress, while the overexpression of *S-adenosyl-Met synthetase 2* increases Arabidopsis tolerance to salt stress [29]. Consistently, our results also show that Met treatment enhances the salt tolerance of WT and *atms2* possibly because a high level of Met might enhance the catalytic reaction from Met to *S*-adenosylmethionine in the one-carbon metabolism pathway (Figure 6a). Therefore, the one-carbon metabolism pathway might play a positive role in plant salt tolerance by regulating the cellular level of Met and *S*-adenosylmethionine, which further affect the methylation level of DNA and histones. Unlike the major contribution of *AtMS1* to Met biosynthesis, the *AtMS2* mutation does not significantly decrease Met content (Figure 6c) [27]. According to the positive role of Met in salt tolerance, it should have been expected that *atms2* has reduced plant salt tolerance. However, our results show that *atms2* enhances plant salt tolerance and that the overexpression of *AtMS2* causes plants to be hypersensitive to salt stress (Figure 4). It seems that AtMS2 might play a predominant role in the repression of plant growth under salt stress in addition to its minor role in Met biosynthesis. Clearly, the repressive role of AtMS2 in *WUS/WOX* expression and plant growth under salt stress might not be related to its catalytic activity and cellular Met content. Therefore, AtMS2 inhibits plant growth under salt stress by repressing meristem activity as a transcriptional factor but fails to increase plant salt tolerance by promoting Met biosynthesis as a metabolic enzyme.

In addition, AtMS1 also interacts with WUS/WOX in the BiFC assay (Figure S5), suggesting that AtMS1 might also be recruited into a transcriptional complex with the WUS/WOX protein. Furthermore, the overexpression of AtMS1:HA protein also causes the hypersensitivity of *35S_pro_::AtMS1:HA* plants to salt stress (Figure S6). These results demonstrate that AtMS1 might also act as a negative factor to repress plant growth under salt stress, although its catalytic product Met plays a positive role in plant salt tolerance. Therefore, AtMS1 has more complicated and contradictory roles in *WUS/WOX* expression, Met metabolism, genome-wide DNA methylation, and plant growth under salt stress. 

The moonlighting function of methionine synthase provides a new signaling pathway for salt stress to sensitively repress meristem activity and plant growth. Although the complete loss of function of *AtMS1* causes a lethal phenotype [27], the loss of function of *AtMS2* confers that plants have enhanced salt tolerance, suggesting that *AtMS2* might be used as a salt tolerance marker for future crop breeding.

## 4. Materials and Methods

### 4.1. Plant Materials

*Arabidopsis thaliana* of Columbia ecotype (Col-0) was used as wild type. The *atms2* mutant (Salk_143628) was obtained from the European Arabidopsis Stock Centre. The *gWUS::GFP:GUS*, *gWOX5::GFP:GUS*, and *gWOX4::GFP:GUS* plants have been described in a recent report [30]. Arabidopsis and tobacco (*Nicotiana benthamiana*) seedlings were cultivated on soil with 120 μmol·m^−2^·s^−1^ white light under long day hours (16 h light/8 h dark) at 22 °C.

### 4.2. Plasmid Construction for Transgenic Plants

The putative 2.841 kb *AtMS2* promoter (−1–−2481 bp) was cloned into *pB7GW2* vector to create *pB7GW2_ AtMS2_pro_* by replacing 35S promoter. The 3.139 kb *AtMS2* coding region and 1.253 kb 3′ downstream region were cloned into the *Xba* I site before *GFP* gene and *Spe* I site behind *GUS* gene in *pDONR201_GFP:GUS* vector, individually, to create *pDONR201*_*gAtMS2:GFP:GUS* for the in-frame fusion of *AtMS2* with *GFP:GUS* gene. The *gAtMS2:GFP:GUS* fusion gene was further cloned into *pB7GW2_AtMS2_pro_* to create *gAtMS2::GFP:GUS* construct using LR clonase (Invitrogen, Carlsbad, CA, USA). The *AtMS2* genomic region containing 3.139 kb coding region and 1.253 kb 3′ downstream region was also cloned into the *pB7GW2_AtMS2_pro_* to create *gAtMS2* construct. *AtMS2* and *AtMS1* cDNA were cloned into *pDONR201* to create *pDONR201_ AtMS2* and *pDONR201_ AtMS1*, respectively. The HA tag and GFP were cloned into the *pDONR201_AtMS2* and the *pDONR201_ AtMS1* to create *pDONR201_AtMS2:HA*, *pDONR201_AtMS1:HA* and *pDONR201-AtMS2:GFP*, respectively. These genes harbored in the *pDONR201* were further cloned into *pK2GW7* to create *35S_pro_::AtMS2:HA*, *35S_pro_::AtMS1:HA*, and *35S_pro_::AtMS2:GFP* constructs, respectively. All constructs were transformed into an *Agrobacterium tumefaciens* PMP90 strain to generate transgenic plants via the floral dip transformation [42].

### 4.3. Protein Purification

The *AtMS2* cDNA was cloned into the *pGEX-4T-1* to express GST:AtMS2 protein. The *AtMS2*, *WUS*, *WOX5*, and *WOX4* were also cloned into the *pMBP-c* vector to express MBP:AtMS2, MBP:WUS, MBP:WOX5, and MBP:WOX4 proteins, respectively. These proteins were expressed in *E. coli* BL21 using 1.0 mM isopropyl β-D-1-thiogalactopyranoside (IPTG) at 16 °C for 12 h and were purified with glutathione resin (Thermo Scientific, Waltham, MA, USA) and amylose resin (New England Bio-Labs, Ipswich, MA, USA), respectively.

### 4.4. In Vitro Pull-Down Assay

The MBP:WUS, MBP:WOX5, MBP:WOX4, and MBP were immobilized on amylose resin and incubated with GST:AtMS2 or GST in a reaction buffer (50 mM Tris-HCl, pH 7.4, 200 mM NaCl, 5.0% glycerol, 1.5% Triton X-100, 10 mM NaF, 1.0 mM Na_3_VO_4_ and 1.0 mM phenylmethylsulfonyl fluoride) for 1.5 h at 4 °C with rotation. The amylose resin was then washed three times and subjected to immunoblot analysis to detect the precipitated GST:AtMS2 protein using an anti-GST antibody (Cell Signaling Technology, Beverly, MA, USA).

### 4.5. Bimolecular Fluorescence Complementation (BiFC) Assays

The *AtMS2* and *AtMS1* cDNA were cloned into the p2YC vector containing C-terminal half of *YFP* to express the fusion proteins of AtMS2-YFP_C_ and AtMS1-YFP_C_, respectively. The vectors expressing WUS-YFP_N_, WOX4-YFP_N_, WOX5-YFP_N_, and WOX1-YFP_N_, individually, have been already described in a recent report [30]. The *35S_pro_::Coilin:RFP* construct was also used to express the nucleus-localized Coilin:RFP protein. The *Agrobacterium tumefaciens* harboring p2YC, p2YN, and *35S_pro_::Coilin:RFP* plasmids were mixed at the ratio of 1:1:1 and infiltrated into *Nicotiana benthamiana* leaves. YFP fluorescence and RFP fluorescence were examined 2 d after the *Agrobacterium* infiltration. 

### 4.6. Fluorescence Scanning

GFP fluorescence, YFP fluorescence, and RFP fluorescence were examined using a confocal laser scanning microscope (Zeiss LSM 780, Jena, Germany) with excitation/emission values of 488 nm/495–545 nm, 513 nm/495–545 nm and 561 nm/578–636 nm, respectively. The laser intensity was usually set between 700 and 800. 

### 4.7. Chromatin Immunoprecipitation (ChIP)

Around 3 g of 3-day-old *35S_pro_::AtMS2:HA* seedlings grown on medium containing 80 mM NaCl was harvested and fixed with 1% formaldehyde (*v*/*w*) under vacuum. ChIP-qPCR was performed with four independent biological samples as described previously [31]. The primers used for ChIP-qPCR are listed in Supplemental Table S1.

### 4.8. Transactivation Assay in Nicotiana Benthamiana

The *WUS_pro_::LUC*, *WOX5_pro_::LUC*, and *WOX4_pro_::LUC* dual-luciferase reporters, as well as *35S_pro_::GFP:WOX5*, *35S_pro_::GFP:WUS*, *35S_pro_::GFP:WOX4*, and *35S_pro_::GFP* constructs, have been described in a recent report [30]. The dual-luciferase reporters in combination with different effectors (*35S_pro_::AtMS2:HA, 35S_pro_::GFP*, *35S_pro_::GFP:WUS, 35S_pro_::GFP:WOX5,* and *35S_pro_::GFP:WOX4*) harbored in *Agrobacterium* were infiltrated into *Nicotiana benthamiana* leaves. The activities of firefly luciferase (FLuc) and renilla luciferase (REN) were examined with 4 independent biological samples using a dual-luciferase assay kit (Promega, Madison, WI, USA) on a Promega GloMax^®^ 20/20 luminometer.

### 4.9. Electrophoretic Mobility Shift Assay (EMSA)

The *WUS* promoter probe (5’-CTAGAATGAATAATAAAAAAAG TGAAAACCGTTTGATCATAA-3’), *WUS* mutant probe (5′-CTAGAATGA AGGGGAAAAAAAGTGAAAACCGTTTGATCATAA-3’), and *WOX5* promoter probe (5’-TGAATTTGTTCTCCTTTAATTTGTGGTGACTCGCACACACTT-3′) labeled with Texas red at 5′ end were used for EMSA assay. The unlabeled probes were used for competition experiments. *WUS*/*WOX5* promoter probe in combination with MBP, MBP:WUS/MBP:WOX5, and MBP:AtMS2 were incubated in a total volume of 20 μL binding buffer (10 mM Hepes-KOH pH 7.8, 20 mM KCl, 10 mM MgCl_2_, 1 mM DTT, 0.1% BSA, 5% glycerol, 0.5% Triton-X100, 20 ng/μL salmon sperm DNA) at 22 °C for 30 min as in a recent report [30]. Then, the reaction products were resolved on 6% polyacrylamide gel and visualized using a ChemiDoc XRS molecular imaging system (Bio-Rad, Hercules, CA, USA).

### 4.10. Co-Immunoprecipitation

The *35S_pro_::GFP:WUS* and *35S_pro_::GFP* in combination with *35S_pro_::AtMS2:HA* were transiently expressed in leaves of 4-week-old *Nicotiana benthamiana* through the *Agrobacterium* infiltration to examine the interaction between AtMS2 and WUS following the protocol described in an earlier report [31]. 

### 4.11. NaCl Treatment

To examine the effects of salt stress on germination ratio, seeds were geminated and grown on Murashige and Skoog (MS) medium containing NaCl at different concentrations in a growth chamber with an irradiance of 120 μmol·m^−2^·s^−1^ white light at 22 °C. For long-term NaCl treatment, 5-day-old WT and *atms2* seedlings were transplanted on soil and watered with 40 mM NaCl at 50 mL per pot once a week.

### 4.12. RNA Isolation and Real-Time Quantitative RT-PCR

Thirty-milligram seedlings were collected to extract total RNA using an Eastep^TM^ Total RNA Super Extraction Kit (Promega, USA). The isolated total RNA around 500 ng was reverse transcribed in a 20 μL reaction mixture using a PrimeScript™II 1st Strand cDNA Synthesis Kit (Takara, Osaka, Japan). The real-time quantitative PCR was performed using a SYBR Premix ExTaq II kit (Takara, Japan) with the CFX96 Touch TM real-time PCR detection system (Bio-Rad, USA). Three replicate assays were performed with independent RNA samples. Actin2 was examined as a reference gene. All primers used for RT-qPCR analysis are listed in Supplemental Table S1.

### 4.13. Measurement of Met Content

Five-day-old 25 mg seedlings were collected and frozen in liquid nitrogen. Samples were ground into powder and resuspended in 300 μL H_2_O. Samples were centrifuged at 12,000× *g* for 20 min at 4 °C; then, the supernatant was transferred into a new tube following freeze drying and resuspended in 100 μL 50% acetonitrile (*v*/*v*) for LC-MS analysis using an ACQUITY UPLC I-class system (Waters, Milford, MA, USA) connected to a QTRAP 6500+ mass spectrometer (AB SCIEX, Framingham, MA, USA). Data were acquired in a multiple reaction monitoring (MRM) model. The survey scan covered an *m*/*z* range of 150.1–104.1. Met at different concentrations (0.32, 1.6, 8, 40, 200 μg × mL^−1^) was also measured for standard curve. Three independent samples were examined for 5-day-old WT and *atms2* mutant grown on MS medium containing 80 mM NaCl or not, individually.

### 4.14. Histochemical Localization of Gus Activity

The histological analysis of β-glucuronidase activity was performed as in a reported protocol [43]. To examine the effects of NaCl treatment on the β-glucuronidase activity in *gWUS::GFP:GUS*, *gWOX5::GFP:GUS*, and *gWOX4::GFP:GUS* plants, at least 60 seedlings were used for GUS staining with incubation in GUS staining solution for 8 h, 4 h, and 6 h, individually. 

### 4.15. Accession Numbers

Sequence data for these genes in this report can be found in the Arabidopsis Genome Initiative with the following accession numbers: *WUS* (At2g17950), *WOX5* (At3g11260), *WOX4* (At1g46480), *AtMS2* (At3g03780), and *AtMS1* (At5g17920).

## Data Availability

The data that support the findings of this study are available within the paper and its Supplementary Materials online.

## Supplementary Materials

The following supporting information can be downloaded at: https://www.mdpi.com/article/10.3390/plants13162224/s1, Figure S1: The accumulation of AtMS2:GFP protein in the nucleus can be triggered by osmotic stress and H_2_O_2_ treatment, but not by ABA treatment. The 5-day-old *35S_pro_::AtMS2:GFP* seedlings were immersed in a control PBS solution (a), 180 µM ABA (b), 2 mM H_2_O_2_ (c), and 0.6 M sorbitol (d) for 24 h before GFP scanning. Scale bars = 200 µm; Figure S2: The expression pattern of *AtMS2* gene in *gAtMS2::GFP:GUS* plant. (a) Diagram of *gAtMS2::GFP:GUS* construct. (b,c) AtMS2:GFP:GUS protein is universally expressed in 3-day-old *gAtMS2::GFP:GUS* seedling (b) and *gAtMS2::GFP:GUS* seedling with true leaves differentiated (c) by GUS staining. (d–f) AtMS2:GFP:GUS protein is universally expressed in silique (d), fluorescent meristem (e) and flower (f) by GUS staining. Scale bar = 0.5 mm (a), 2.0 mm (b–d), 5.0 mm (e), and 1.5 mm (f); Figure S3: The expression of *AtMS2* mRNA in *atms2* mutant by RT-PCR. The *AtMS2* mRNA at full length was detected in WT seedling, but not *atms2* mutant, by RT-PCR at 36 amplification cycles. *Actin2* was examined as a reference gene by RT-PCR at 24 amplification cycles. Four independent samples were examined; Figure S4: The phenotype of root meristem of WT, *atms2* and *35Spro::AtMS2:GFP* plants. The *35Spro::AtMS2:GFP* plant has a shorter root apical meristem in comparison with WT and *atms2* plants. The root of 5-day-old seedlings was stained by propidium iodide. Scale bar = 100 µm; Figure S5: AtMS1 protein interacts with WUS/WOX in the nucleus. (a–g) Bimolecular fluorescence complementation in *Nicotiana benthamiana* epidermal cells. AtMS1:YFP_C_ protein in combination with WUS:YFP_N_ (a), WOX5:YFP_N_ (b), and WOX4:YFP_N_ (c), respectively, was colocalized with Coilin:RFP protein in nucleus. WOX1:YFP_N_, and Chloramphenicol Acetyltransferase (CHL):YFP_N_ were used in control assays (d–g). Scale bar = 40 µm; Figure S6: Overexpression of AtMS1 represses plant growth under salt stress. (a,b) The growth phenotype of 5-day-old WT and *35S_pro_::AtMS1:HA* seedlings on media containing 80 mM NaCl in comparison with control media (a). Scale bar = 1.0 cm; Table S1: Primers used in ChIP analysis and RT-qPCR analysis.
