# Supplementary material for: Methionine Synthase 2 Represses Stem Cell Maintenance of Arabidopsis thaliana in Response to Salt Stress
Source: Plants (Basel). 2024 Aug 10;13(16):2224. doi: 10.3390/plants13162224 (PMC11359516; doi:10.3390/plants13162224)
Supplement: Supplementary file 1 [file plants-13-02224-s001.zip › plants-3095954-supplementary.pdf]

Supplemental data

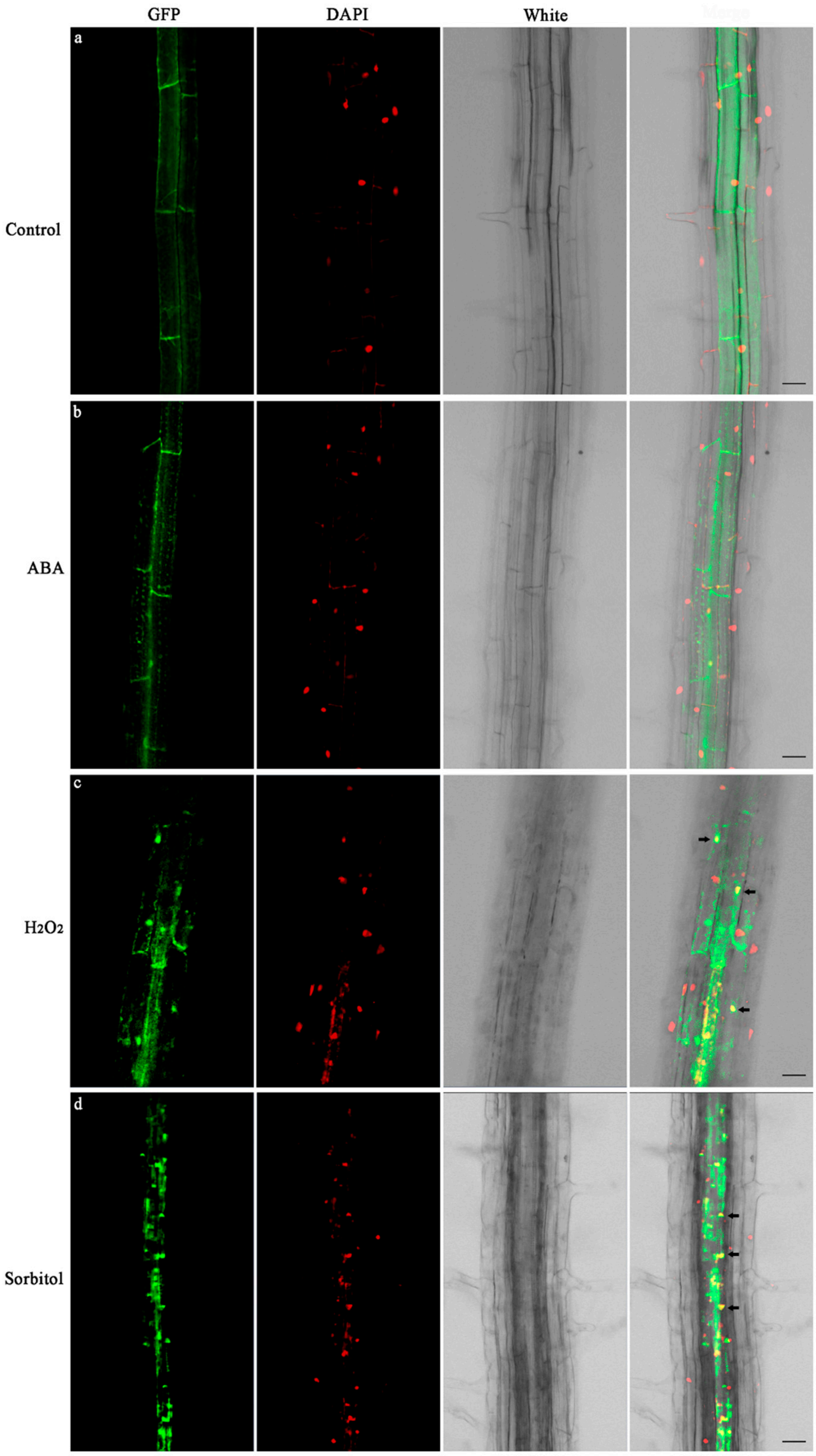

Figure S1. The accumulation of AtMS2:GFP protein in the nucleus can be triggered by osmotic stress and H_2O_2 treatment, but not by ABA treatment. The 5-day-old *35S$_{pro}$::AtMS2:GFP* seedlings were immersed in a control PBS solution (a), 180 µM ABA

(b), 2 mM H_2O_2 (c), and 0.6 M sorbitol (d) for 24 h before GFP scanning. Scale bars = 200μm.

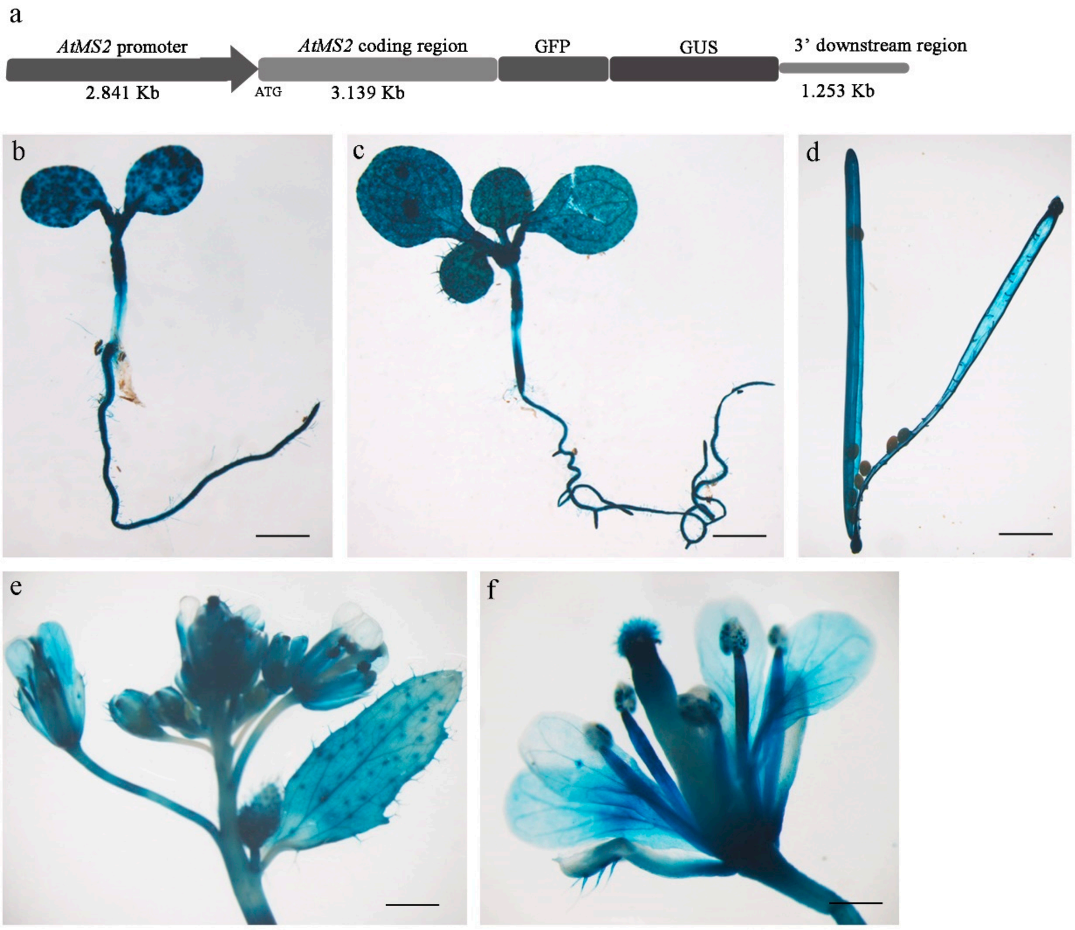

Figure S2. The expression pattern of *AtMS2* gene in *gAtMS2::GFP:GUS* plant. (a) Diagram of *gAtMS2::GFP:GUS* construct. (b,c) AtMS2:GFP:GUS protein is universally expressed in 3-day-old *gAtMS2::GFP:GUS* seedling (b) and *gAtMS2::GFP:GUS* seedling with true leaves differentiated (c) by GUS staining. (d–f) AtMS2:GFP:GUS protein is universally expressed in silique (d), fluorescent meristem (e) and flower (f) by GUS staining. Scale bar = 0.5 mm (a), 2.0 mm (b–d), 5.0 mm (e), and 1.5 mm (f).

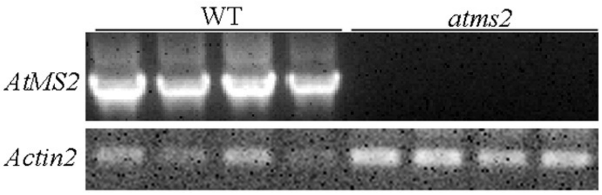

Figure S3. The expression of *AtMS2* mRNA in *atms2* mutant by RT-PCR. The *AtMS2* mRNA at full length was detected in WT seedling, but not *atms2* mutant, by RT-PCR at 36 amplification cycles. *Actin2* was examined as a reference gene by RT-PCR at 24 amplification cycles. Four independent samples were examined.

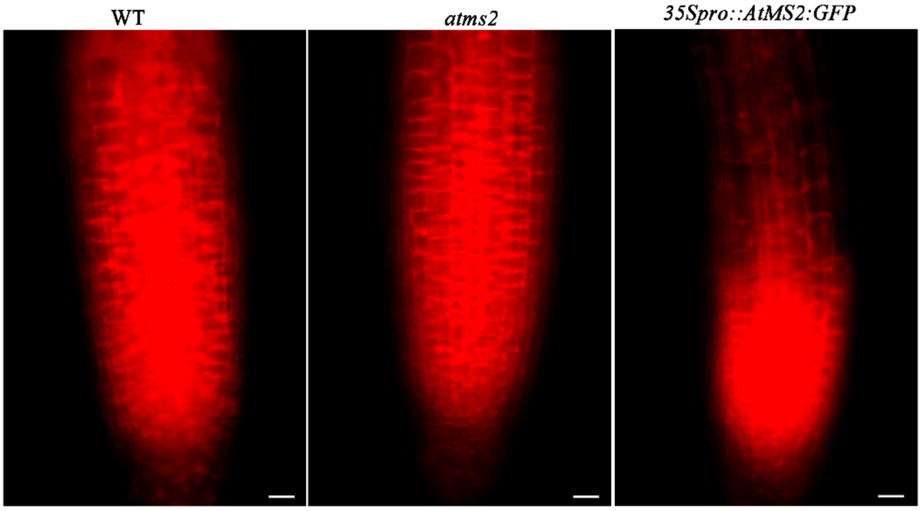

Figure S4. The phenotype of root meristem of WT, *atms2* and *35Spro::AtMS2:GFP* plants. The *35Spro::AtMS2:GFP* plant has a shorter root apical meristem in comparison with WT and *atms2* plants. The root of 5-day-old seedlings was stained by propidium iodide. Scale bar = 100 μm.

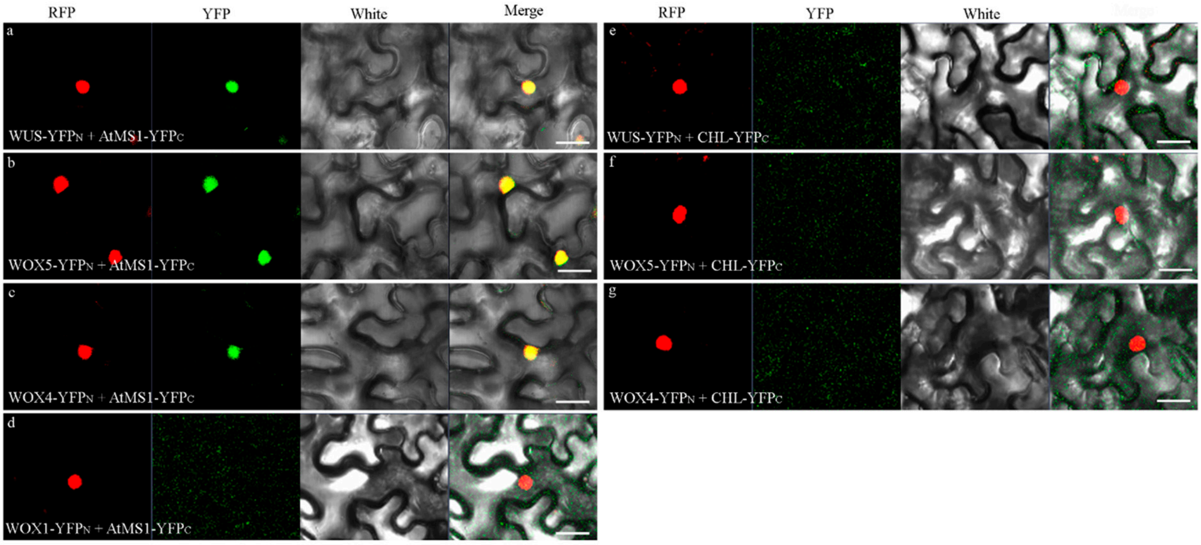

Figure S5. AtMS1 protein interacts with WUS/WOX in the nucleus. (a–g) Bimolecular fluorescence complementation in *Nicotiana benthamiana* epidermal cells. AtMS1:YFP$_{C}$ protein in combination with WUS:YFP$_{N}$ (a), WOX5:YFP$_{N}$ (b), and WOX4:YFP$_{N}$ (c), respectively, was colocalized with Coilin:RFP protein in nucleus. WOX1:YFP$_{N}$, and Chloramphenicol Acetyltransferase (CHL):YFP$_{N}$ were used in control assays (d–g). Scale bar = 40 μm.

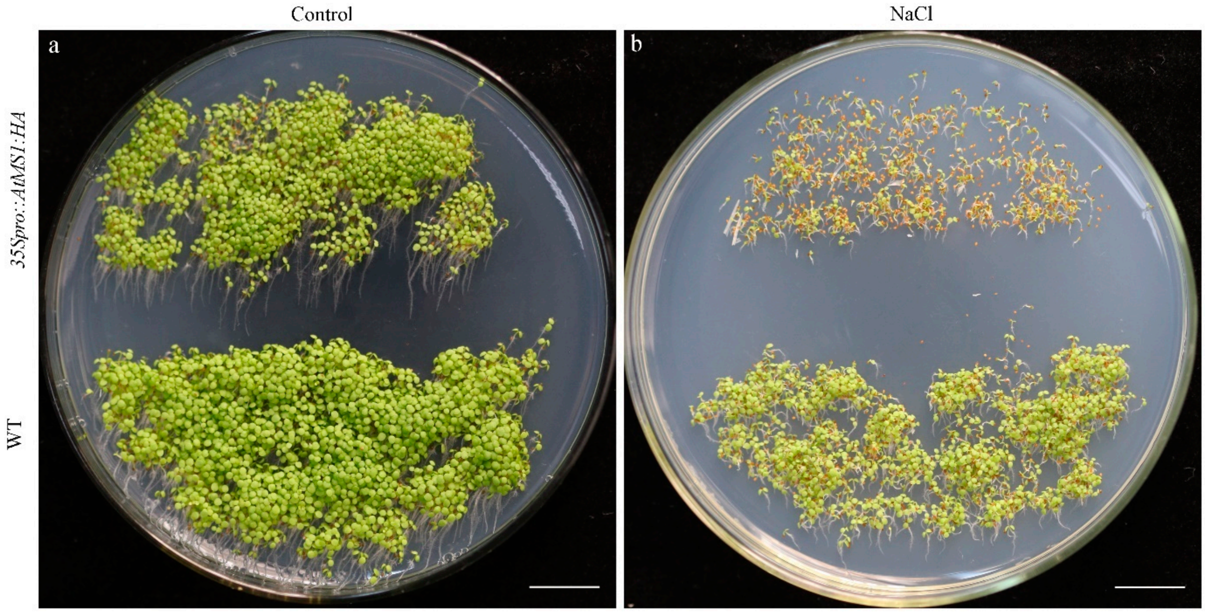

Figure S6. Overexpression of AtMS1 represses plant growth under salt stress. (a,b) The growth phenotype of 5-day-old WT and *35S*$_{pro}$*::AtMS1:HA* seedlings on media containing 80 mM NaCl in comparison with control media (a). Scale bar = 1.0 cm.

Table S1. Primers used in ChIP analysis and RT-qPCR analysis.

Primers used in ChIP analysis

Amplification region	Forward primer	Reverse primer
WUS promoter	5'-CATGACCATAAGGAAGGAAAAATGT-3'	5'-ACCCATACACACAGAAAACAAAAC-3'
WOX5 promoter	5'-CCCCATGTATGCTTGTGAACAATTAATA-3'	5'-GTCAAGCGTGTAACTTTTTCCTAAGTAA-3'
WOX4 promoter	5'-GACTTTCCACCTTCATAACTTTGATATC-3'	5'-GCAGAACCTCAATTTCCCAAGTAAT-3'
WUS exon	5'-GTGGTGGCGATGCTTATCTG-3'	5'-GCGCAAGGGCGAACTTCCGA-3'

Primers used in RT-qPCR analysis

Genes	Forward primer	Reverse primer
WUS	5'-TCACCATCATCACGGTGTTC-3'	5'-AGAACAGTCTTGTTCCATAGA-3'
WOX5	5'-CTATTGGTTTCAGAATCATAAGGCTA-3'	5'-TCAAAATCAATGGAGATTTTACGA-3'
WOX4	5'-CCTCCGGCGTCACTTCAG-3'	5'-GGGTTCCACCTTGTCCCTC-3'
CLV3	5’-GACTTTCCAACCGCAAGATG-3’	5'-TCATGTAGTCCTAAACCCTTCGT -3'
ACTIN2	5'-GGTGGTTCCATTCTTGCTTC-3'	5'-GAAACGCAGACGTAAGTAAAAAC-3'
